# Potential Involvement of Reactive Oxygen Species in the Bactericidal Activity of Eugenol against *Salmonella* Typhimurium

**DOI:** 10.3390/pathogens13100899

**Published:** 2024-10-14

**Authors:** Mohammed Aljuwayd, Elena G. Olson, Asim Zahoor Abbasi, Michael J. Rothrock, Steven C. Ricke, Young Min Kwon

**Affiliations:** 1 Cell and Molecular Biology Program, University of Arkansas, Fayetteville, AR 72701, USA; maljuway@uark.edu (M.A.); asima@uark.edu (A.Z.A.); ykwon@uark.edu (Y.M.K.); 2 College of Medical Applied Sciences, The Northern Border University, Arar 91431, Saudi Arabia; 3 Meat Science and Animal Biologics Discovery Program, Department of Animal and Dairy Sciences, University of Wisconsin, Madison, WI 53706, USA; egolson2@wisc.edu; 4 United States Department of Agriculture, Agricultural Research Service, Athens, GA 30605, USA; michael.rothrock@usda.gov; 5 Department of Poultry Science, University of Arkansas System Division of Agriculture, Fayetteville, AR 72701, USA

**Keywords:** antimicrobials, phytochemicals, eugenol, *Salmonella*, reactive oxygen species

## Abstract

There is an increasing need to develop alternative antimicrobials to replace currently used antibiotics. Phytochemicals, such as essential oils, have garnered significant attention in recent years as potential antimicrobials. However, the mechanisms underlying their bactericidal activities are not yet fully understood. In this study, we investigated the bactericidal activity of eugenol oil against *Salmonella* enterica serovar Typhimurium (*S*. Typhimurium) to elucidate its mechanism of action. We hypothesized that eugenol exerts its bactericidal effects through the production of reactive oxygen species (ROS), which ultimately leads to cell death. The result of this study demonstrated that the bactericidal activity of eugenol against *S*. Typhimurium was significantly (*p* < 0.05) mitigated by thiourea (ROS scavenger) or iron chelator 2,2′-dipyridyl, supporting the hypothesis. This finding contributes to a better understanding of the killing mechanism by eugenol oil.

## 1. Introduction

Foodborne diseases are among the most significant public health challenges worldwide [1]. In the United States alone, these diseases affect millions of people, causing substantial morbidity and mortality, and costing billions of dollars annually, and are of global concern as well [2,3,4]. Only non-typhoidal *Salmonella* (NTS) serovars can be transferred from animals to humans and detected in a wide range of animal hosts [5,6]. Therefore, controlling *Salmonella* in food production and processing is critically important [6]. The emergence of *Salmonella* strains resistant to third-generation cephalosporins and other antibiotics, particularly those encoded on plasmids, is a growing concern as it can lead to the further dissemination of antibiotic resistance [5,7,8]. Thus, research into alternative antimicrobials to limit *Salmonella* dissemination is urgently needed [6]. Medicinal plants have become a promising source of antimicrobial compounds for treating bacterial infections, with studies showing the bactericidal activities of phytochemicals such as eugenol [9,10].

Eugenol oil is an essential oil derived from plants such as clove, nutmeg, cinnamon, and basil, and mainly contains eugenol (C_10_H_12_O_2_) [10]. It showed antibacterial activity against *Helicobacter pylori* [11]. Jafri et al. [12] found that a 1% eugenol solution reduces the viability of *Candida albicans* and *Streptococcus mutans* in biofilms Another study showed its antibacterial effects against *Salmonella* Typhi [13]. Eugenol disrupts bacterial cell membranes and increases permeability [13,14,15]. In pathogens such as *Candida albicans,* this leads to cytoplasmic leakage and superoxide anions (ROS) accumulation [15]. The ROS accumulation causes oxidative stress, damaging proteins, lipids, and DNA. In *Escherichia coli*, eugenol treatment increased divalent cations and membrane depolarization, enhancing its antibacterial activity [14].

Thiourea is a scavenger of hydroxyl radicals (•OH), one of the most reactive and damaging ROS [16]. Thiourea can react with hydroxyl radicals and hydrogen peroxide, neutralizing them and preventing oxidative damage [16]. Additionally, 2,2′-dipyridyl inhibits ROS formation by chelating iron ions, thereby preventing reactions that generate ROS, such as the Fenton and Haber–Weiss reactions [16]. By chelating these metal ions, it prevents them from participating in oxidation–reduction reactions that generate ROS. By binding to iron, 2,2′-dipyridyl prevents iron from catalyzing this reaction, thereby reducing the production of hydroxyl radicals and other ROS [16]. Korobov et al. [17] used 2,2′-dipyridyl to show the bactericidal effect of the bacteriocin warnerin on *Staphylococcus cohnii* was via ROS accumulation. Similarly, in the current study, thiourea and 2,2′-dipyridyl were used to confirm the role of ROS in the bactericidal mechanism of eugenol. We hypothesized that eugenol would decrease *S*. Typhimurium survival while the addition of thiourea or 2,2′-dipyridyl would increase *Salmonella* survival as compared to eugenol alone.

## 2. Materials and Methods

### 2.1. Experimental Design

*Salmonella* Typhimurium strain 14028s obtained from ATCC (Manassas, VA, USA) and used in this study was streaked on Luria–Bertani (LB) agar (Fisher Scientific; Waltham, MA, USA) plates and incubated aerobically overnight at 37 °C. The following day, a single colony was picked, inoculated in LB, and incubated at the same atmosphere and temperature. The next day, the culture was reinoculated in new LB broth until it reached an optical density of 0.6 at 600 nm (4.8 × 10^8^ CFU/mL). The culture was subsequently separated into four groups: a control group (no treatment) and three different treatment groups with 4 replications per treatment. The first treatment group consisted of cell cultures treated with 1% eugenol (Thermo Fisher Scientific, Waltham, MA, USA). In the second group, 150 mM thiourea (VWR Chemicals, Sanborn, NY, USA) was added along with 1% eugenol. In the third group, 500 µM Dip (2,2 dipyridyl; VWR Chemicals) was added along with 1% eugenol.

**Figure 1:**
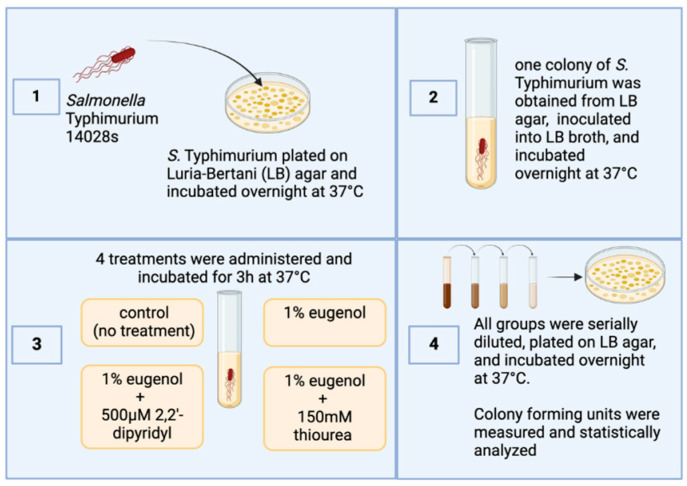
Overview of the experimental design of the current study. Figure generated by Biorender (Olson, E. (10-1-2024), BioRender.com/w22n378, accessed on 1 October 2024).

The treatments were then incubated aerobically at 37 °C. All groups were serially diluted and spread-plated on LB agar. The plates were incubated aerobically at 37 °C overnight. The next day, colony-forming units (CFU/mL) were measured and recorded for statistical analysis (Figure 1). The Institutional Biosafety Committee (IBC) at the University of Arkansas, Fayetteville, AR, USA, approved all laboratory experiments involving this pathogen (Biosafety Level 2).

### 2.2. Statistical Analysis

To assess the main effect of the model, we utilized Analysis of Variance (ANOVA) followed by Student’s *t*-test to determine whether a significant difference existed between the control and treatment groups. A reduction was considered significant for treatment groups if *p* < 0.05 compared to the control. All data were analyzed using (JMP^®^, Version JMP pro-17, SAS Institute Inc., Cary, NC, 1989–2023, USA).

## 3. Results and Discussion

**Figure 2:**
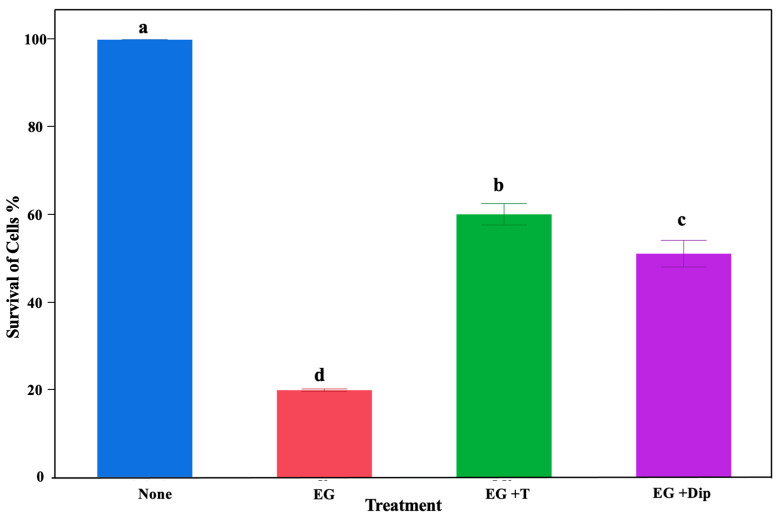
Effect of different treatments on *Salmonella* growth. The bar graph shows inhibitory effects of different treatments on controlling *Salmonella* Typhimurium abundance. The no-treatment group was used as a control (None). The treatment groups included cultures treated with 1% eugenol (EG), 1% eugenol + 150 mM thiourea (EG + T), and 1% eugenol + 2,2′dipyridyl (EG + Dip). Letters a–d denote significant differences.

To demonstrate the significant role of hydroxyl radicals in the lethal effects of eugenol on *S*. Typhimurium, we used the iron-chelating 2,2′-dipyridyl and ROS-scavenging thiourea. There was a significant effect of the treatments in the current study (*p* < 0.0001). Particularly, all treatments resulted in a significant reduction (*p* < 0.001) in *Salmonella* abundance compared to the control (no treatment) (Figure 2). The highest reduction was observed in the group treated with 1% eugenol, resulting in an over 80% *Salmonella* reduction. The groups treated with 150 mM thiourea + 1% eugenol and 2,2′-dipyridyl + 1% eugenol showed around 40% and 49% reductions, respectively (Figure 2).

Eugenol’s antibacterial properties are attributed to its free hydroxyl groups, which disrupt bacterial cell membranes, increase ROS concentrations, or affect enzymatic activities. This study demonstrated that both thiourea and 2,2′-dipyridyl significantly increased the survival of *S*. Typhimurium, indicating the role of hydroxyl radicals in the bactericidal process of eugenol. Existing research supports this study’s findings on the antimicrobial properties of eugenol. Eugenol has been shown to be effective against a range of pathogens, including fungal species and both Gram-positive and Gram-negative bacteria [18,19,20,21,22]. Previous studies have highlighted the effectiveness of eugenol in reducing *Salmonella* in human food and animal feed, aligning with the observed significant reduction in *Salmonella* abundance in the current in vitro study [9,23,24,25,26,27,28]. The involvement of hydroxyl radicals in the antibacterial activity of eugenol has been noted in prior studies. Free hydroxyl groups are known to disrupt bacterial cell membranes and increase ROS, leading to bacterial cell death [19,20,22]. The use of ROS scavengers such as thiourea and iron chelators like 2,2′-dipyridyl to demonstrate the role of ROS in eugenol’s bactericidal action is consistent with existing research on the mechanism of action of plant-derived antimicrobials [19,20,22]. This study underscores the potential of eugenol as an anti-*Salmonella* phytochemical. This aligns with ongoing efforts to alter gastrointestinal microbiota using various strategies, including probiotics and prebiotics [29,30].

Incorporating eugenol in food materials or water as a botanical antimicrobial could enhance *Salmonella* mitigation strategies, reducing the bacterial load during or before food processing. As a food or animal feed additive, eugenol could be integrated into processed food or animal feed to continuously manage bacterial populations, leveraging its antimicrobial properties to maintain gut health and reduce the incidence of infections [28,31,32,33]. Understanding the role of hydroxyl radicals in eugenol’s mechanism can aid in optimizing its use. Strategies that avoid or mitigate ROS scavengers in feed could enhance the efficacy of eugenol as an antimicrobial agent.

Combining eugenol with agents that increase ROS can heighten oxidative stress in pathogens, leading to higher mortality by overwhelming bacterial defenses. Targeting multiple bacterial pathways (e.g., membrane integrity by eugenol and metabolic pathways by other antimicrobials) enhances pathogen control and reduces survival chances [34]. Using eugenol with prebiotics and probiotics fosters a healthier gut microbiota balance: eugenol reduces pathogen load, while prebiotics and probiotics support beneficial bacteria, improving gut health [35]. This approach prevents pathogen colonization through mechanisms such as competitive exclusion by probiotics and direct killing by eugenol, significantly reducing pathogen burden in the gastro-intestinal environment. Synergistic antimicrobial effects lower pathogen *Salmonella* colonization within the gut environment, reducing disease outbreaks and improving overall health. A healthier gut environment, bolstered by eugenol and other antimicrobials, enhances the immune response in the gut, providing further protection against infections [36].

The exact mechanisms by which eugenol reduces *Salmonella* need further investigation, especially in the in vivo models. It is crucial to explore how eugenol targets various *Salmonella* serovars and strains in the animal models and assess its effects on gut health. Eugenol possesses antimicrobial potency, which makes this a promising candidate for controlling *Salmonella* in the gut environment, but the generation of ROS from eugenol at different pH levels may need to be considered [37]. It is also crucial to explore how eugenol affects the non-*Salmonella* gut microbiota, particularly beneficial bacteria, for determining its antimicrobial range. Further research into the molecular processes by which eugenol targets *Salmonella* is needed to develop practical applications for public health and food safety.

## 4. Conclusions

This study highlights the potent antimicrobial properties of eugenol, particularly its efficacy in reducing *Salmonella* abundance. This research underscores the role of hydroxyl radicals in the bactericidal action of eugenol, supported by the observed reduction in *Salmonella* when ROS scavengers were introduced. This aligns with previous findings on the disruption of bacterial cell membranes and increased ROS concentration due to eugenol’s free hydroxyl groups. The significant reduction in *Salmonella* abundance, especially with the 1% eugenol treatment, demonstrates its potential as a botanical ingredient that can be added in different food matrices for inhibiting foodborne pathogen growth [28,38]. Further research is necessary to fully elucidate potential additional mechanisms by which eugenol targets different *Salmonella* serovars and strains, and its impact on non-*Salmonella* gut microbiota. Understanding these mechanisms will be crucial for optimizing eugenol’s application in managing *Salmonella* infections and developing effective strategies to improve public health outcomes.

## Data Availability

Any inquiries about the original data can be directed to the corresponding author.

## References

[1] Grace D. (2023). Burden of foodborne disease in low-income and middle-income countries and opportunities for scaling food safety interventions. Food Secur..

[2] Painter J.A., Hoekstra R.M., Ayers T., Tauxe R.V., Braden C.R., Angulo F.J., Griffin P.M. (2013). Attribution of foodborne illnesses, hospitalizations, and deaths to food commodities by using outbreak data, United States, 1998–2008. Emerg. Infect. Dis..

[3] Scharff R.L. (2012). The economic burden of foodborne illness in the United States. J. Food Prot..

[4] Majowicz S.E., Musto J., Scallan E., Angulo F.J., Kirk M., O’Brien S.J., Jones T.F., Fazil A., Hoekstra R.M., International Collaboration on Enteric Disease “Burden of Illness” Studies (2010). The global burden of nontyphoidal *Salmonella* gastroenteritis. Clin. Infect. Dis..

[5] Galán-Relaño Á., Díaz A.V., Lorenzo B.H., Gómez-Gascón L., Rodríguez M.M., Jiménez E.C., Rodríguez F.P., Márquez R.J.A. (2023). *Salmonella* and salmonellosis: An update on public health implications and control strategies. Animals.

[6] Mkangara M. (2023). Prevention and control of human *Salmonella* enterica infections: An implication in food safety. Int. J. Food Sci..

[7] Raut R., Maharjan P., Fouladkhah A.C. (2023). Practical preventive considerations for reducing the public health burden of poultry-related salmonellosis. Int. J. Environ. Res. Public Health.

[8] Jeon H.Y., Seo K.W., Kim Y.B., Kim D.K., Kim S.W., Lee Y.J. (2019). Characteristics of third-generation cephalosporin-resistant *Salmonella* from retail chicken meat produced by integrated broiler operations. Poult. Sci..

[9] Bajpai V.K., Baek K.-H., Kang S.C. (2012). Control of *Salmonella* in foods by using essential oils: A review. Food Res. Int..

[10] Khalil A.A., ur Rahman U., Khan M.R., Sahar A., Mehmood T., Khan M. (2017). Essential oil eugenol: Sources, extraction techniques and nutraceutical perspectives. RSC Adv..

[11] Elbestawy M.K.M., El-Sherbiny G.M., Moghannem S.A. (2023). Antibacterial, antibiofilm and anti-inflammatory activities of eugenol clove essential oil against resistant *Helicobacter pylori*. Molecules.

[12] Jafri H., Banerjee G., Khan M.S.A., Ahmad I., Abulreesh H.H., Althubiani A.S. (2020). Synergistic interaction of eugenol and antimicrobial drugs in eradication of single and mixed biofilms of Candida albicans and Streptococcus mutans. AMB Express.

[13] Devi K.P., Arif Nisha S., Sakthivel R., Pandian S.K. (2010). Eugenol (an essential oil of clove) acts as an antibacterial agent against *Salmonella* typhi by disrupting the cellular membrane. J. Ethnopharmacol..

[14] Jeyakumar G.E., Lawrence R. (2021). Mechanisms of bactericidal action of eugenol against *Escherichia coli*. J. Herb. Med..

[15] Shahina Z., Ndlovu E., Persaud O., Sultana T., Dahms T.E.S. (2022). *Candida albicans* reactive oxygen species (ROS)-dependent lethality and ROS-independent hyphal and biofilm inhibition by eugenol and citral. Microbiol. Spectr..

[16] Karash S., Kwon Y.M. (2018). Iron-dependent essential genes in *Salmonella* Typhimurium. BMC Genom..

[17] Korobov V.P., Lemkina L.M., Polyudova T.V. (2022). The mechanism of antibacterial action of the lantibiotic warnerin. Microbiology.

[18] Marchese A., Barbieri R., Coppo E., Orhan I.E., Daglia M., Izadi M., Abdollahi M., Nabavi S.M., Ajami M. (2017). Antimicrobial activity of eugenol and essential oils containing eugenol: A mechanistic viewpoint. Crit. Rev. Microbiol..

[19] Braga P., Sasso M.D., Culici M., Alfieri M. (2007). Eugenol and thymol, alone or in combination, induce morphological alterations in the envelope of Candida albicans. Fitoterapia.

[20] Das B., Mandal D., Dash S.K., Chattopadhyay S., Tripathy S., Dolai D.P., Dey S.K., Roy S. (2016). Eugenol provokes ROS-mediated membrane damage-associated antibacterial activity against clinically isolated multidrug-resistant *Staphylococcus aureus* strains. Infect. Dis. Res. Treat..

[21] Ulanowska M., Olas B. (2021). Biological properties and prospects for the application of eugenol—A review. Int. J. Mol. Sci..

[22] Di Pasqua R., Betts G., Hoskins N., Edwards M., Ercolini D., Mauriello G. (2007). Membrane toxicity of antimicrobial compounds from essential oils. J. Agric. Food Chem..

[23] López-Romero J.C., Valenzuela-Melendres M., Juneja V.K., García-Dávila J., Camou J.P., Peña-Ramos A., González-Ríos H. (2018). Effects and interactions of gallic acid, eugenol and temperature on thermal inactivation of *Salmonella* spp. in ground chicken. Food Res. Int..

[24] Nair D.V., Manjankattil S., Peichel C., Martin W., Donoghue A.M., Venkitanarayanan K., Kollanoor-Johny A. (2023). Effect of plant-derived antimicrobials, eugenol, carvacrol, and β-resorcylic acid against *Salmonella* on organic chicken wings and carcasses. Poult. Sci..

[25] Kollanoor-Johny A., Mattson T., Baskaran S.A., Amalaradjou M.A., Babapoor S., March B., Valipe S., Darre M., Hoagland T., Schreiber D. (2012). Reduction of *Salmonella enterica* serovar Enteritidis colonization in 20-day-old broiler chickens by the plant-derived compounds *trans*-cinnamaldehyde and eugenol. Appl. Environ. Microbiol..

[26] Zhao X., Wei S., Tian Q., Peng W., Tao Y., Bo R., Liu M., Li J. (2022). Eugenol exposure in vitro inhibits the expressions of T3SS and TIF virulence genes in *Salmonella* Typhimurium and reduces its pathogenicity to chickens. Microb. Pathog..

[27] Zhao X., Zheng S., Wei S., Tian Q., Tao Y., Bo R., Liu M., Li J. (2022). The protective effect and potential mechanisms of eugenol against *Salmonella* in vivo and in vitro. Poult. Sci..

[28] Olaimat A.N., Al-Holy M.A., Osaili T.M., Abughoush M.H., Al-Nabulsi A.A., Alawneh M., Deseh L., Abazeed B., Shqair R., Mutlaq S. (2024). Antimicrobial activity of eugenol and carvacrol against *Salmonella* enterica and *E. coli* O157:H7 in falafel paste at different storage temperatures. Int. J. Food Microbiol..

[29] Zeng H., De Reu K., Gabriël S., Mattheus W., De Zutter L., Rasschaert G. (2021). *Salmonella* prevalence and persistence in industrialized poultry slaughterhouses. Poult. Sci..

[30] Clavijo V., Flórez M.J.V. (2018). The gastrointestinal microbiome and its association with the control of pathogens in broiler chicken production: A review. Poult. Sci..

[31] Petrovska B.B. (2012). Historical review of medicinal plants’ usage. Pharmacogn. Rev..

[32] Mussarat S., Ali R., Ali S., Mothana R.A., Ullah R., Adnan M. (2021). Medicinal animals and plants as alternative and complementary medicine in southern regions of Khyber Pakhtunkhwa, Pakistan. Front. Pharmacol..

[33] Borges A., Abreu A.C., Dias C., Saavedra M.J., Borges F., Simões M. (2016). New perspectives on the use of phytochemicals as an emergent strategy to control bacterial infections including biofilms. Molecules.

[34] Didehdar M., Chegini Z., Shariati A. (2022). Eugenol: A novel therapeutic agent for the inhibition of *Candida* species infection. Front. Pharmacol..

[35] Fujisawa S., Murakami Y., Gupta S.C., Prasad S., Aggarwal B.B. (2016). Eugenol and its role in chronic diseases. Drug Discovery from Mother Nature.

[36] Song F., Liu J., Zhao W., Huang H., Hu D., Chen H., Zhang H., Chen W., Gu Z. (2020). Synergistic effect of eugenol and probiotic *Lactobacillus plantarum* Zs2058 against *Salmonella* infection in C57bl/6 mice. Nutrients.

[37] Atsumi T., Iwakura I., Fujisawa S., Ueha T. (2001). Reactive oxygen species generation and photo-cytotoxicity of eugenol in solutions of various pH. Biomaterials.

[38] Luo K., Kang S., Guo M., Shen C., Wang L., Xia X., Lü X., Shi C. (2022). Evaluation of the antibacterial mechanism and biofilm removal effect of eugenol on *Vibrio vulnificus* and its application in fresh oysters. Food Biosci..

